# The KDEL trafficking receptor exploits pH to tune the strength of an unusual short hydrogen bond

**DOI:** 10.1038/s41598-020-73906-3

**Published:** 2020-10-09

**Authors:** Zhiyi Wu, Simon Newstead, Philip C. Biggin

**Affiliations:** Department of Biochemistry, South Parks Road, Oxford, OX1 3QU UK

**Keywords:** Computational biophysics, Membrane trafficking

## Abstract

The endoplasmic reticulum (ER) is the main site of protein synthesis in eukaryotic cells and requires a high concentration of luminal chaperones to function. During protein synthesis, ER luminal chaperones are swept along the secretory pathway and must be retrieved to maintain cell viability. ER protein retrieval is achieved by the KDEL receptor, which recognises a C-terminal Lys-Asp-Glu-Leu (KDEL) sequence. Recognition of ER proteins by the KDEL receptor is pH dependent, with binding occurring under acidic conditions in the Golgi and release under conditions of higher pH in the ER. Recent crystal structures of the KDEL receptor in the apo and peptide bound state suggested that peptide binding drives the formation of a short-hydrogen bond that locks the KDEL sequence in the receptor and activates the receptor for COPI binding in the cytoplasm. Using quantum mechanical calculations we demonstrate that the strength of this short hydrogen bond is reinforced following protonation of a nearby histidine, providing a conceptual link between receptor protonation and KDEL peptide binding. Protonation also controls the water networks adjacent to the peptide binding site, leading to a conformational change that ultimately allows the receptor-complex to be recognized by the COPI system.

## Introduction

In eukaryotic cells newly synthesised proteins destined for secretion pass from the endoplasmic reticulum (ER) to the Golgi^1^. Luminal ER chaperones and quality control enzymes are normally associated with these proteins and are trafficked along with their substrates to the Golgi apparatus. To maintain the required pool of folding chaperones in the ER, it is essential that these proteins are retrieved^2^. ER protein retrieval is mediated by the KDEL receptor (KDELR), which recognises escaped proteins in the Golgi and mediates their return to the ER via COPI vesicles^3,4^. The KDELR recognizes ER proteins through a C-terminal ER retrieval sequence (ERS), consisting of Lys-Asp-Glu-Leu (KDEL)^5,6^, although variations to the canonical sequence exist^7,8^. Binding of the KDEL sequence to the receptor is pH dependent^9^. In the more acidic environment of the Golgi lumen the receptor forms a stable complex with the KDEL bearing cargo proteins. The activated receptor then signals across the Golgi membrane to recruit COPI and initiate retrograde trafficking to the ER. Following retrieval, the higher pH in the ER results in deprotonation of the receptor and release of the KDEL peptide and associated cargo protein, whereupon the receptor is cycled back to the Golgi via COPII vesicles^10,11^.

**Figure 1:**
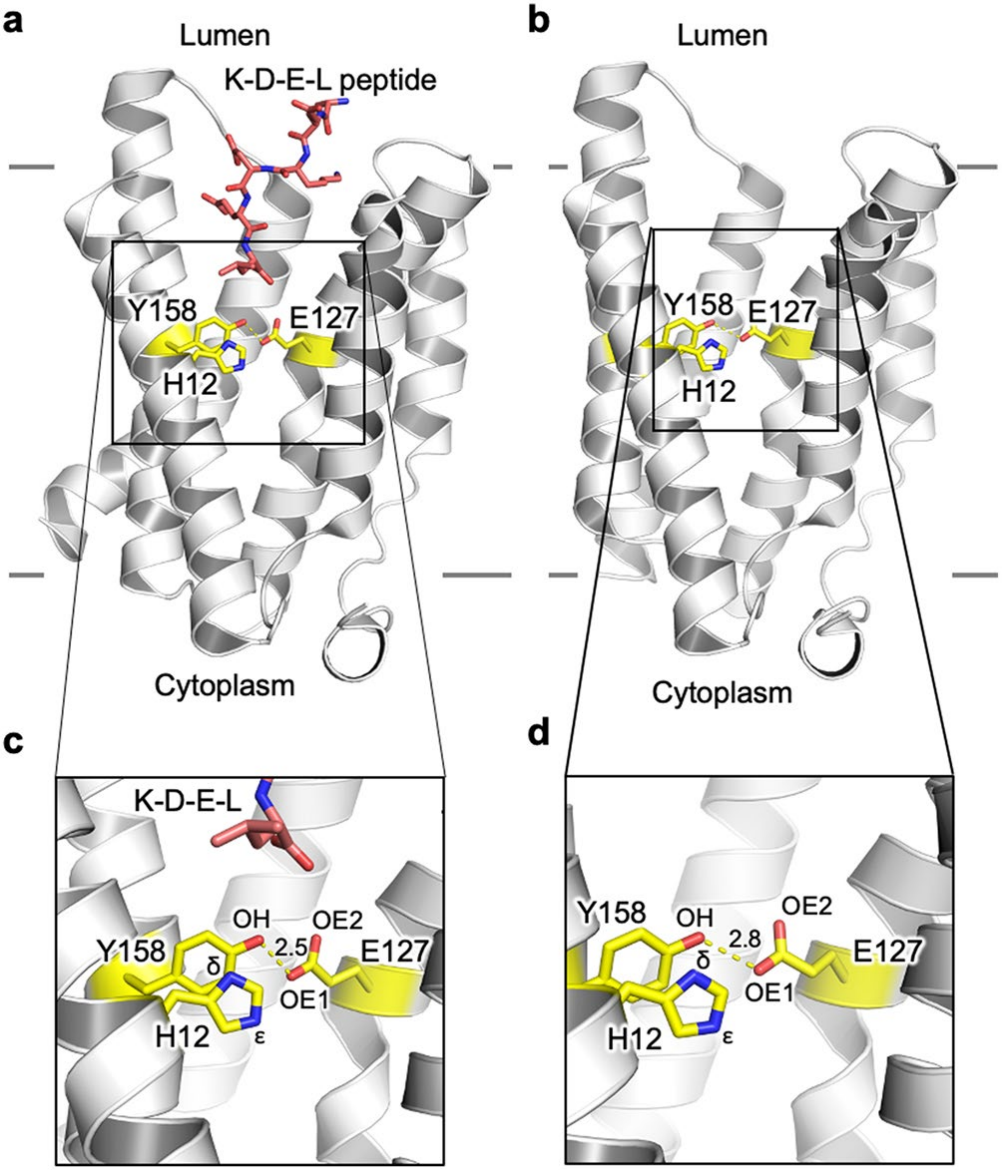
Structures of the KDEL receptor used in this study. (**a**,**c**) KDEL peptide bound (PDB: 6i6h) and (**b**,**d**) the Apo (PDB: 6i6b) state. The hydrogen bond between E127 and Y158 is slightly shorter when peptide is bound at 2.5 Å (**c**) compared to 2.8 Å in the apo form (**d**). Atom labels used within this study are also shown along with the position of H12, which has previously be shown to act as the pH sensor. The approximate location of the membrane is shown with solid gray lines.

Although it was discovered over 2 decades ago, the molecular mechanism of pH-dependent binding by the KDELR remains stubbornly elusive^12^. Recently the crystal structure of the KDELR has been solved in both KDEL bound (pH 6.0) and Apo state (pH 9.0)^13^ (Fig. 1). The structures revealed the conformational changes that occur upon signal peptide binding, which alter the electrostatic surface of the receptor on the cytoplasmic side of the membrane that likely mediates the interaction of the receptor with either COPI or COPII to allow for KDEL dependent recycling of the receptor within the secretory pathway. In vitro binding and cellular retrieval assays highlighted an essential role for a conserved histidine (H12) at the base of the signal peptide binding pocket. Due to the protonatable nature of this residue within the physiological range of the secretory pathway, it was proposed that this side chain may form the pH sensor for the KDEL retrieval system. This histidine is located adjacent to an unusually short hydrogen bond formed between a conserved tyrosine (Y158) and glutamate (E127), which functions to lock the receptor in an active state following binding of the ERS. The importance of both the H12 and the hydrogen bond has already been shown via mutation where H12A, E127A, E127Q, Y158F all abolish the peptide binding capability^13^. Furthermore, the trinity of this hydrogen bond (H12, E127 and Y158) is strictly conserved across multiple species^13^. However, the structures raise several interesting questions pertinent to our understanding of this system, including: What is the role of the short hydrogen bond and how might this interaction influence the behavior of the receptor? Can the energetic contribution of this be quantified? How does the protonation state of H12 influence the energetics of the system in a way that reflects the functional cycle of the KDEL receptor? Is the protonation state of H12 dependent of the presence of the short hydrogen bond and vice versa?

Using a combination of molecular dynamics (MD) and quantum mechanics (QM) calculations we explored the role of this conserved histidine side chain in pH sensing, and the influence this has on the physical properties of the short hydrogen bond located at the core of the receptor. Our results show that protonation of H12 is essential for the formation of the short hydrogen bond, and thus plays an essential role in stabilising the active state of the receptor. We also show that the presence of the short hydrogen-bond appears to dictate whether a continuous water cavity can form, which our simulations suggest may play an important role in the structural changes that result in signaling the recruitment of either the COPI or COPII coatomer proteins.

## Results

### The strength of the E127⋯H—Y158 H-bond depends on the protonation state of H12

Simulation of a short-hydrogen bond is particularly problematic for simple molecular mechanics based representations due to the inability of the force-fields to properly capture the electronic nature of such bonds. Thus, we used QM methods to assess the energetics of the E127-Y158 hydrogen bond based on the coordinates from the crystal structure (PDB: 6I6H). However, as the crystal structures do not indicate where the proton is most likely to be, we initially computed the energy of QM geometry optimized structures (SI Text 1, SI Fig. 1, SI Table 1). These calculations confirmed the expectation that the most favourable position for the proton in all states is close to the tyrosine hydroxyl oxygen. The calculations also suggested that the energy barrier for the hydrogen to move to/from the nearest sidechain oxygen of E127 was likely to be very small (a few kT).

**Table 1:** Properties of the E127⋯H—Y158 H-bond.

	KDEL-bound			Apo		
	HID	HIE	HIP	HID	HIE	HIP
	Interaction free energy between E127 and Y158 (kcal/mol)					
∆G	− 30.33	− 27.30	− 31.37	− 20.55	− 17.4	− 15.83
	The core-valence bifurcation index (CVBi)					
CVBi	− 0.23	− 0.20	− 0.26	− 0.03	− 0.02	0.04
	Quantum theory of atoms in molecules (QTAIM)					
H(r)	− 0.031	− 0.028	− 0.034	− 0.002	− 0.001	− 0.002
V(r)/G(r)	− 1.53	− 1.48	− 1.58	− 1.09	− 1.06	− 1.09
H(r)/ρ(r)	− 0.39	− 0.36	− 0.41	− 0.06	− 0.05	− 0.07

HID and HIE indicate the proton in neutral histidine is positioned the δ or ε nitrogen respectively (see Fig. 1). HIP indicates that both nitrogens are protonated leading to positively charged histidine. CVBi is the Core Valence Bifurcation index. H(r) is the total energy density, V(r) is the potential energy density, G(r) is the Lagrangian kinetic energy and p(r) is the electron density. Details of the interpretation of the parameters reported for the QTAIM analysis are given in the SI.

Given the potential role of H12 in mediating pH response in the receptor, we postulated that the protonation state of H12 might affect the strength of the E127⋯H—Y158 H-bond, which in turn would be expected to influence the ability of the receptor to lock the KDEL receptor in the activated state. We explored this aspect by examining the change in free energy required to transition from the KDEL-bound state to the Apo state under different protonation states of H12.

As is shown in Table 1, the free energy difference of transition from KDEL-bound to Apo state is highest when H12 is protonated (HIP: − 15.83 − (− 31.37) = 15.54 kcal/mol) than the neutral form of histidine (HID: − 20.55 − (− 30.33) = 9.78 kcal/mol; HIE: − 17.4 − (− 27.30) = 9.9 kcal/mol). Thus, 5.7 kcal/mol more free energy is required for the transition when H12 is protonated.

Having shown that the free energy penalty of transition is greatly enhanced when the histidine is protonated, we then assessed to what extent the short hydrogen bond is itself influenced by the protonation state of H12. The crudest way of estimating H-bond strength is via the length of the bond between the hydrogen and hydrogen bond donor R(D–H)^14^. As shown in Table 1, the R(D–H) distances in the KDEL-bound state are all longer than that in the Apo state, suggesting a stronger hydrogen bond in the KDEL-bound state compared with the Apo state. Furthermore, in the KDEL-bound state, the R(D–H) is longest when H12 is protonated, suggesting that protonation strengthens the hydrogen bond. Such an effect is not observed in the Apo state, where the R(D–H) is the same regardless of the protonation state of the histidine.

A more accurate estimate of H-bond strength can be obtained via the QM methods known as Core-Valence Bifurcation Index (CVBi)^15^ and quantum theory of atoms in molecules QTAIM^16^ (see Methods and SI). All these methods give a consistent result that the H-bond is stronger in the KDEL-bound state compared with the Apo state. For example, the CVBi values are significantly more negative for the KDEL-bound state than the Apo, and the total energy density (H(r)), is more negative in the KDEL-bound than in the Apo state. More details on the interpretation of the QTAIM values are given in SI Text 2. Furthermore, we note that in the KDEL-bound state, this hydrogen bond is further enhanced by the protonation of the histidine. This effect is not seen in the Apo state, where the strength of the H-bond seems to be independent of the protonation state of H12.

Though we have demonstrated that the protonated H12 strengthens the interaction between E127 and Y158 via enhancement of the hydrogen bond, it is still unclear as to how much the protonation state of histidine would affect the strength of the hydrogen bond. A very short H-bond, such as that observed in the KDEL-bound state, is indicative of a low-barrier H-bond. To test if this hydrogen bond is indeed a low-barrier hydrogen bond, we computed the potential energy landscape of transferring the proton from the hydrogen bond donor (Y158) to the hydrogen bond acceptor (E127). If we consider the Apo state first (Fig. 3a), this shows a typical profile whereby there is a large (~ 20 kcal/mol) energy barrier to transfer the proton from the Y158 to E127. Moreover, there is very little effect from the protonation of H12. The profile for the KDEL-bound state is very different (Fig. 3b) and the protonation state of H12 can have a significant effect. When the histidine is deprotonated, the energy difference between the global energy minimum (proton bound to the donor) and the local energy minimum (proton bound to the acceptor) is very high (~ 8 kcal/mol for HIE and ~ 7 kcal/mol for HID). However, when the histidine is protonated (HIP), this energy difference shrinks to only 4 kcal/mol. Thus, in the KDEL-bound state, only when H12 is protonated, can the hydrogen bond be classified as a low-barrier hydrogen bond and is consistent with our hypothesis that the protonated histidine strengthens the hydrogen bond in the KDEL-bound state.

**Figure 2:**
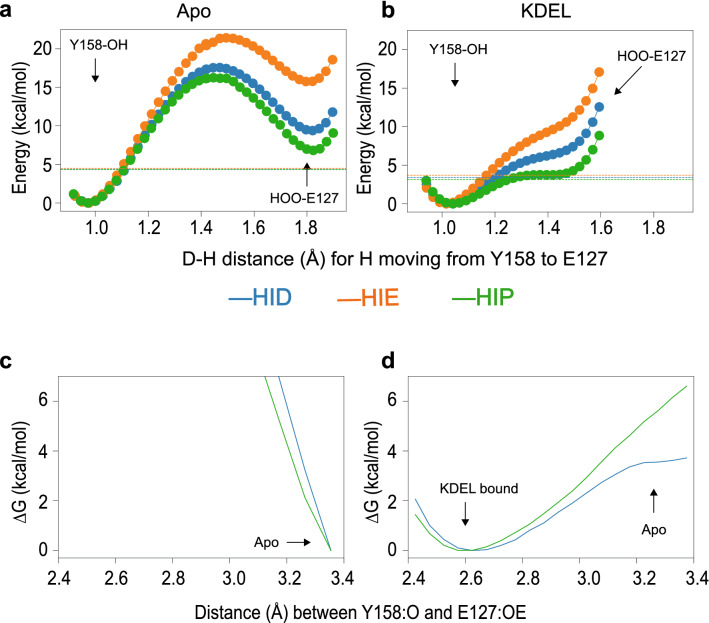
QM estimate of energy of proton transfer from the Y158 to E127. (**a**) In the Apo state, a very large barrier (~ 20 kcal/mol) is observed during the proton transfer, which is not observed in the KDEL-bound state. (**b**) In the KDEL-bound state, the energy difference for the proton movement is smaller than the ZPE (dashed line) when the histidine is protonated (green). The energy difference is greatly increased when histidine is deprotonated (orange and blue). (**c**,**d)** QM/MM based energy landscape of separating the hydrogen bond. **c**. In the Apo state, the short hydrogen bond is prohibited by the larger energy barrier. (**d**) In the KDEL-bound state, 6.5 kcal/mol of energy is required to break the hydrogen bond when histidine is protonated (green line), while only 3.5 kcal/mol is required when histidine is deprotonated (blue line).

Though the QM calculations showed that the protonated histidine allows the criteria of a low-barrier hydrogen bond to be met, a potential issue is that these calculations are performed under vacuum conditions. Thus, to address this issue we also performed QM/MM calculations to compute the free energy barrier of stretching the hydrogen bond in both KDEL-bound and Apo states with both deprotonated (HID) and protonated histidine (HIP). As shown in Fig. 2c, in the Apo state, the calculations demonstrate that the short hydrogen bond is unable to form regardless of the protonation state. In the KDEL-bound state (Fig. 2d) however, when the histidine is deprotonated (HID), some initial resistance is met when separating the H-bond, which quickly plateaus, resulting in only ~ 3.5 kcal/mol for the transition. However, when the histidine is protonated, the resistance is ever increasing, and the free energy penalty is around 6.5 kcal/mol when reaching the same distance as that observed in the Apo state.

### The short hydrogen bond influences the affinity of H12 for protons

**Figure 3:**
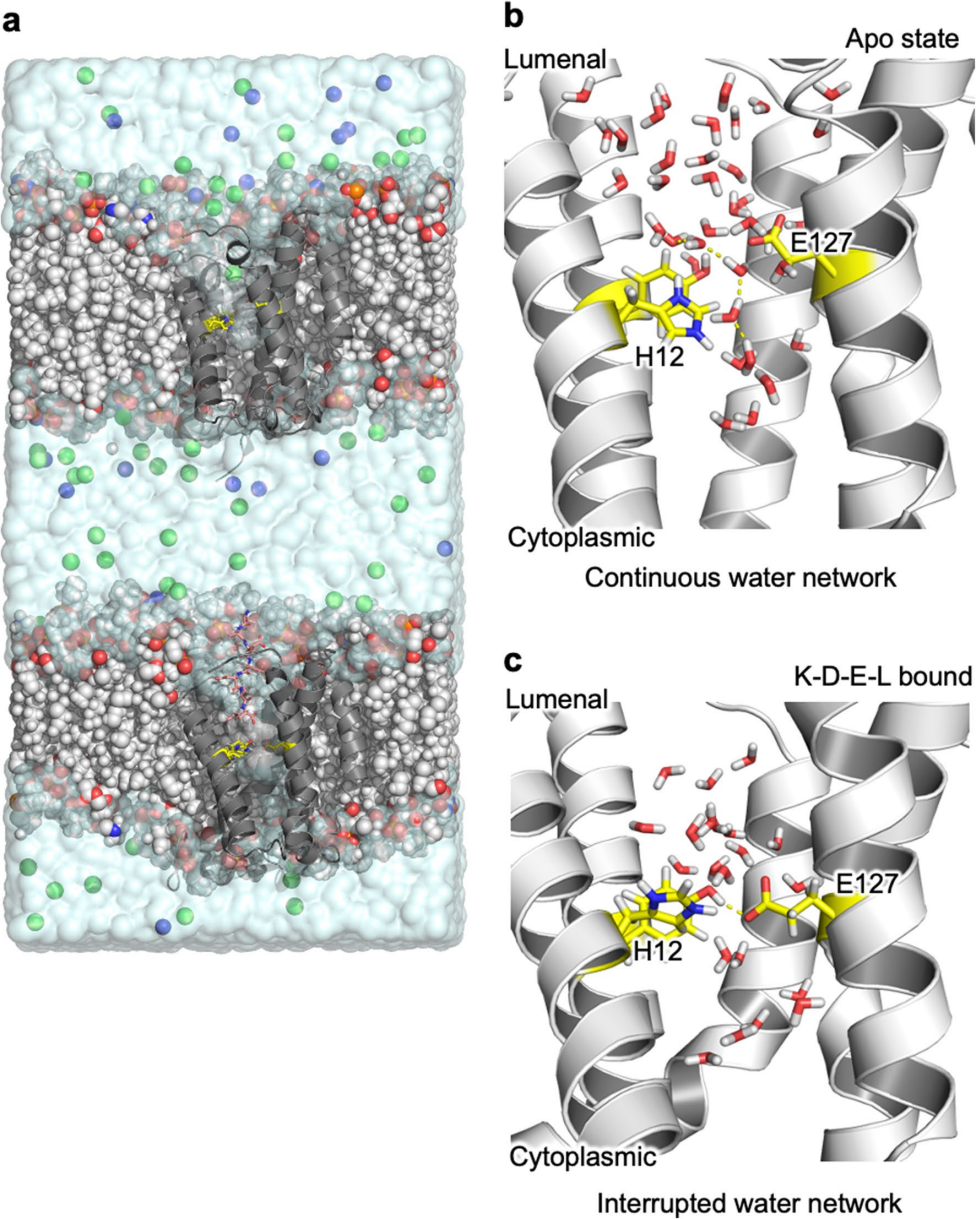
Influence of the short H-bond on H12 affinity using classical molecular mechanics based MD (**a**) shows the simulation box used, which is a double bilayer system to simultaneously add/remove a proton from H12 in each KDEL receptor. Na^+^ and Cl^−^ ions are represented as blue and green spheres respectively and the lipid bilayer is drawn as vdW spheres. (**b**) In the Apo state and when the H12 is protonated, a continuous water network linking the lumenal and cytoplasmic pockets is observed as highlighted by yellow dashed lines. (**c**) In the KDEL-bound state, the presence of the short hydrogen bond prohibits the formation of the water network. H12, E127 and Y158 are drawn as yellow sticks in all figure parts.

Since protonated histidine stabilised the low-barrier hydrogen bond in the KDEL-bound state, but had no effect on the hydrogen bond in the Apo state, the reciprocal effect (i.e. that the short H-bond in the KDEL-bound state should stabilize a protonated H12, but no such effect will be present in the Apo state), should also be true. We employed a double bilayer approach (Methods and Fig. 3) to perform alchemical transformations simultaneously in the Apo and KDEL-bound states to compute the difference in proton affinity in these different states under two different regimens; one where the short hydrogen bond is imposed via harmonic restraint throughout the whole process and the other where there are no restraints to maintain the short H-bond distance (see Methods). The calculations show that under the conditions of the short H-bond, the protonation is more favourable in the KDEL-bound state compared to the Apo state, by ~ 3.5 kcal/mol (Table 2). However, when the short H-bond is not constrained, this difference is reduced to ~ 2 kcal/mol (Table 2). Control calculations performed on histidines elsewhere on the protein (H11 and H90) suggest that any local variation of protonation is likely to be small, of the order of 0.5 kcal/mol.

**Table 2:** ∆∆G of (in kcal/mol) of deprotonation* of H12 in the KDEL-bound and protonation** of H12 in apo state under different conditions.

Condition	Run 1	Run 2	Run 3
Short H-bond imposed	3.555	3.787	3.156
No H-bond restraint	1.992	1.996	2.176
H90 (control—no restraint)	0.847		
H11 (control— no restraint)	0.435		

*To or from** a neutral histidine with the proton on the δ carbon (HID).

To further understand the molecular mechanism behind this short H-bond mediated protonation event, we examined the dynamics of these systems in more detail. In all the transformations performed, H12 was extremely stable (SI Table 2) in terms of its conformation and no ring flipping was observed. The presence of the short H-bond enhances the stability of H12, which is further increased when H12 is protonated.

We then examined the local water environment around the short H-bond. Topologically, the KDEL receptor resembles a transporter with both extracellular and intracellular pockets that contain water molecules^13^. The short H-bond forms a constriction point between these two pockets. In the Apo state (ie in the absence of the short H-bond), the two water pockets are joined by a chain of water molecules (Fig. 3b). In the KDEL-bound state, on the other hand, the communication between two water pockets is interrupted by the presence of the short H-bond (Fig. 3c). The short H-bond appears to “squeeze the waters” away and further into the pockets either side. On the face of it, and considered in isolation, this may appear quite surprising because the protonated histidine in conjunction with the short H-bond might be expected to facilitate coalescence of water in this region.

**Figure 4:**
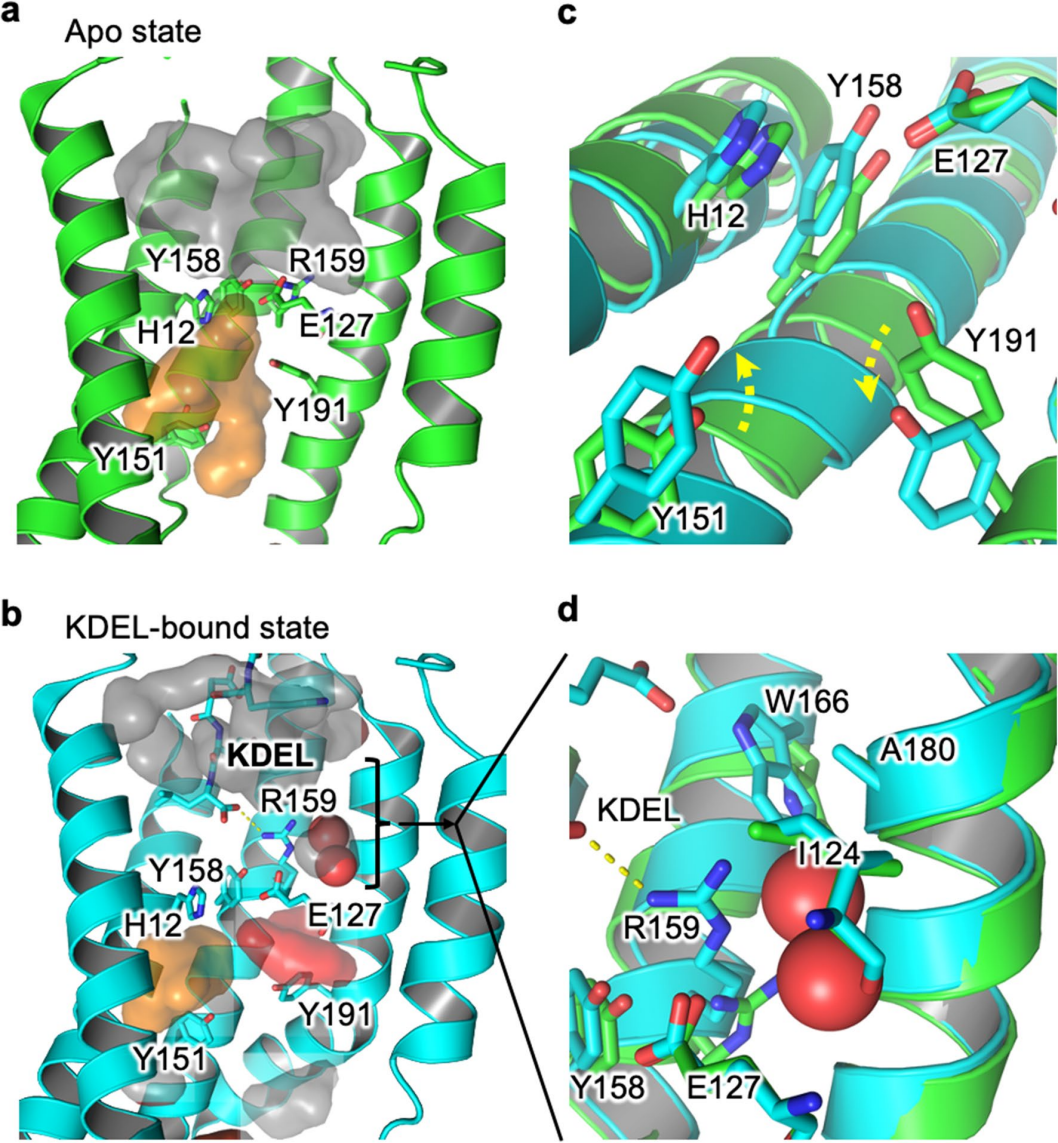
Influence of KDEL-binding upon water pockets (**a**) Apo state (green cartoon) there are two large fully-solvated pockets to the lumenal (grey) and cytoplasmic sides (orange). They are almost, but not quite, connected. (**b**) Upon binding of KDEL the conformational changes in the protein (cyan cartoon) result in the shrinkage of both original water pockets and the formation of an additional pocket between E127 and Y191 (red). (**c**) Summarises the key conformational changes that occur when moving from the Apo (green) to KDEL-bound state (cyan). Y151 extends into the water pocket (orange surface in **a**,**b**), reducing its size while Y191 retracts away to help the formation of the new water pocket (red surface in **b**). (**d**) KDEL binding creates a zone of ordered waters, where the largest cavity is occupied by two waters surrounded by a hydrophobic pocket formed from I124, W166 and A180.

However, there are other conformational differences that exist between the Apo and KDEL-bound states that influence the size and behaviour of water molecules within these pockets (Fig. 4a,b). These structural re-arrangements also affect the water environment around H12. Specifically, KDEL binding leads to an extension of Y151 and the retraction of Y191 (Fig. 4c). The retraction of Y191 allows an additional water pocket to form (Fig. 4b). The extension of the Y151 shrinks the water pocket solvating the NƐ of H12. KDEL-binding leads to a dramatic change in the nature of the water pocket. Most notably, there is a zone of about 9 Å, between the short H-bond and the hydrogen bond formed between R159 and the C-terminus of the KDEL-peptide, where the largest cavity only permits two water molecules to exist. This cavity is a relatively hydrophobic pocket created by I124, W166 and A180 (Fig. 4d). This is in sharp contrast to the Apo state (Fig. 4a), where the binding pocket is filled with disordered water and is almost touching the water pocket under the hydrogen bond formed by E127 and Y158.

**Figure 5:**
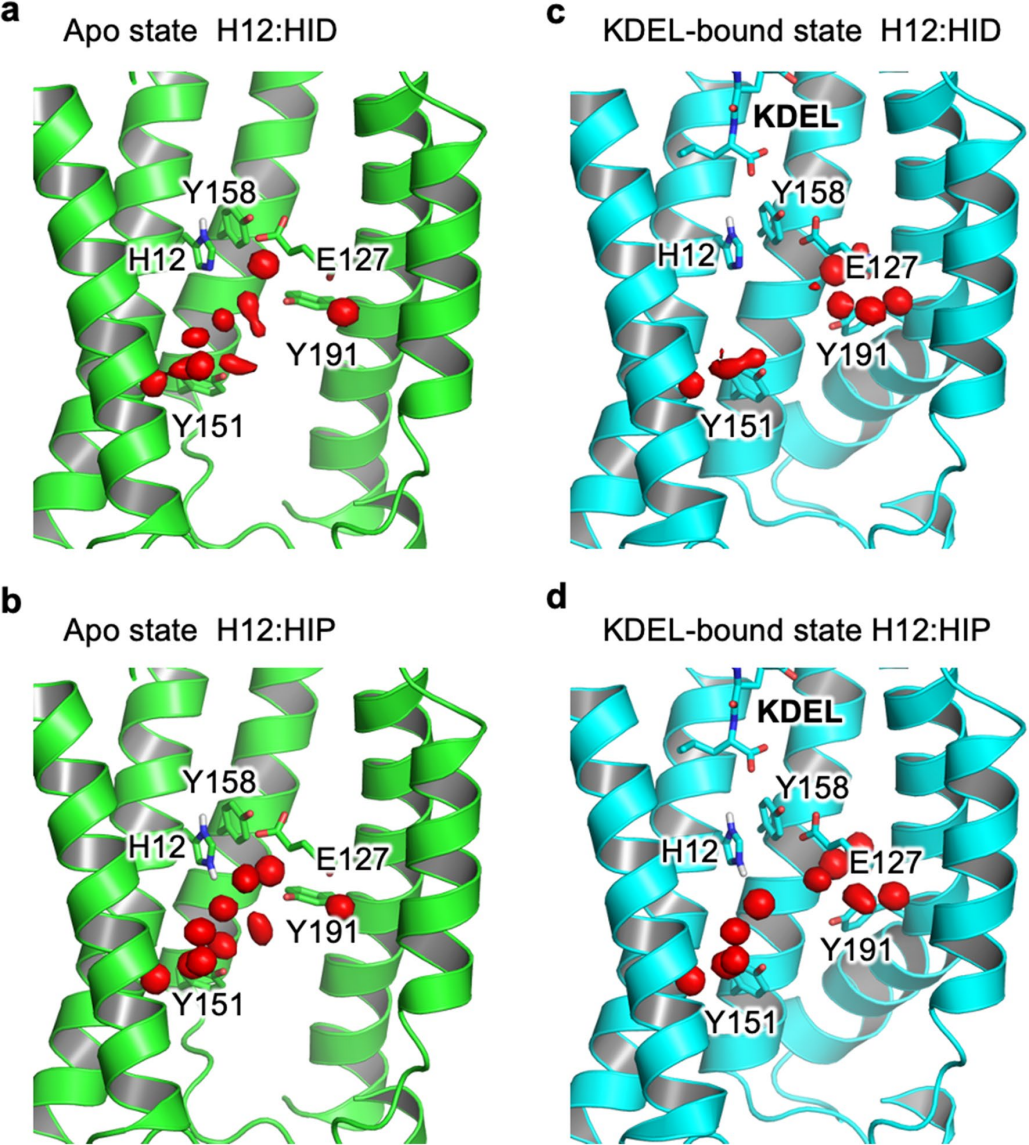
GCMC calculations of water occupancy. (**a**,**b**) The water network that lies on the cytoplasmic side of H12 are not influenced by the protonation state of H12. (**c**,**d**) in contrast, in the KDEL-bound state, deprotonated H12 is unable to participate in any water interactions (**c**) whereas protonated H12 (**d**) enables a continuous water-wire to be formed with Y151.

To further understand how the size and shape of these pockets influences the water behaviour, we undertook Grand Canonical Monte Carlo (GCMC) simulations (see Methods) to investigate preferred water occupancies in the different states and under the different protonation states of H12. The GCMC calculations (Fig. 5) in the KDEL-bound system revealed very different water behaviour in the pocket (towards the cytoplasmic side) depending on the H12 protonation state. In the KDEL-bound state (Fig. 5c), five ordered water molecules solvated the protonated H12 and connect it to Y151. When H12 is deprotonated the Nε is no longer solvated. In the Apo state, due to the larger volume of the pockets, waters can reorient themselves to solvate both the protonated and deprotonated H12 (Fig. 5c,d). Thus, via control of the size of the water pocket, only protonated histidine can be stably solvated in the KDEL-bounds state, giving rise to the 3.5 kcal/mol difference.

The energy difference is maximal when the short H-bond is imposed (Table 2). In the alchemical transformation simulations (Fig. 3 and Table 2), if the short H-bond is not imposed the free energy difference reduces down to ~ 2 kcal/mol, and this reflects the formation of sporadic water wires which resemble the water dynamics observed in the Apo state. All of these calculations are consistent with our hypothesis that the protonated histidine strengthens the hydrogen bond and that the transition from KDEL-bound state to apo state is 3 kcal/mol less when H12 is protonated.

## Discussion

The coordinates of the oxygen atoms in the E127–Y158 in the crystal structure of the peptide bound KDEL receptor are unusual in their close separation of 2.5 Angstroms and suggest the formation of a short hydrogen bond between these two side chains. Although there are numerous precedencies for such observations^17^, we must still consider that the structure could represent something non-physiological (ie. a conformation induced by packing effects) or that the interpretation of the electron density of the oxygen atoms is incorrect. The latter is highly unlikely, because the electron density is very good for this region of the map (even when contoured at 3.5 σ, the signal for the oxygens is very clear). However, we cannot completely rule out that the close positioning of E127 and Y158 in the peptide-bound structure is an artefact of the crystal lattice. Thus, rather than to set out to prove the existence of short H-bond per se, we set out to ask what the consequences of such a short H-bond might be and indeed how it itself might be influenced by its own environment.

Short H-bonds, where the donor–acceptor distance is typically shorter than 2.7 Å are often found in the active sites of enzymes^18^. In that context, such bonds have been described as “low-barrier H-bonds”^19,20^. A feature of such bonds is that the proton is more readily delocalized between the donor and acceptor atoms and the resulting H-bond is predicted to be about 10–20 kcal/mol stronger than normal H-bonds^21,22^. The results here confirm the initial postulation that in the KDEL-bound state there is an unusually short H-bond between E127 and Y158. The fact that it is a tyrosine is also rather interesting as a recent study revealed an unexpected enhancement of tyrosine in such interactions^23^. The (QM) potential energy surface of proton transfer (Fig. 2) confirms that in the apo state there is a large and expected barrier to move the proton away from its preferred position. In the KDEL-bound state, the profile adopts a single well with a shoulder. At this distance, (2.5 Å between donor and acceptor oxygens), the result is entirely consistent with other reports of short H-bond proton potential energy surfaces (review recently by^17^). Furthermore, the calculations suggest the ease of proton-sharing is significantly increased if H12 is protonated. H12, which has already been identified as the proton sensor^13^, would be expected to be protonated in the Golgi, where it also binds to KDEL-bearing cargo proteins.

The above calculations suggest that the presence (and strength) of the short H-bond will be influenced by the protonation state of H12. We also investigated whether presence or absence of a short H-bond will influence the likely protonation state of H12. The results confirm that the presence of the short H-bond favours protonation of H12. Moreover, this appears to be linked to the dynamics of the surrounding water molecules (Fig. 3b,c). Inspection of trajectories suggests that in the KDEL-bound state, with a short H-bond present and H12 protonated, the water pockets either side of the H-bond are not connected (Fig. 4). Moreover, the ability of H12 in the protonated state, but not the deprotonated state, to allow an ordered water network of five waters to form explains the energetic differences (Fig. 5).

**Figure 6:**
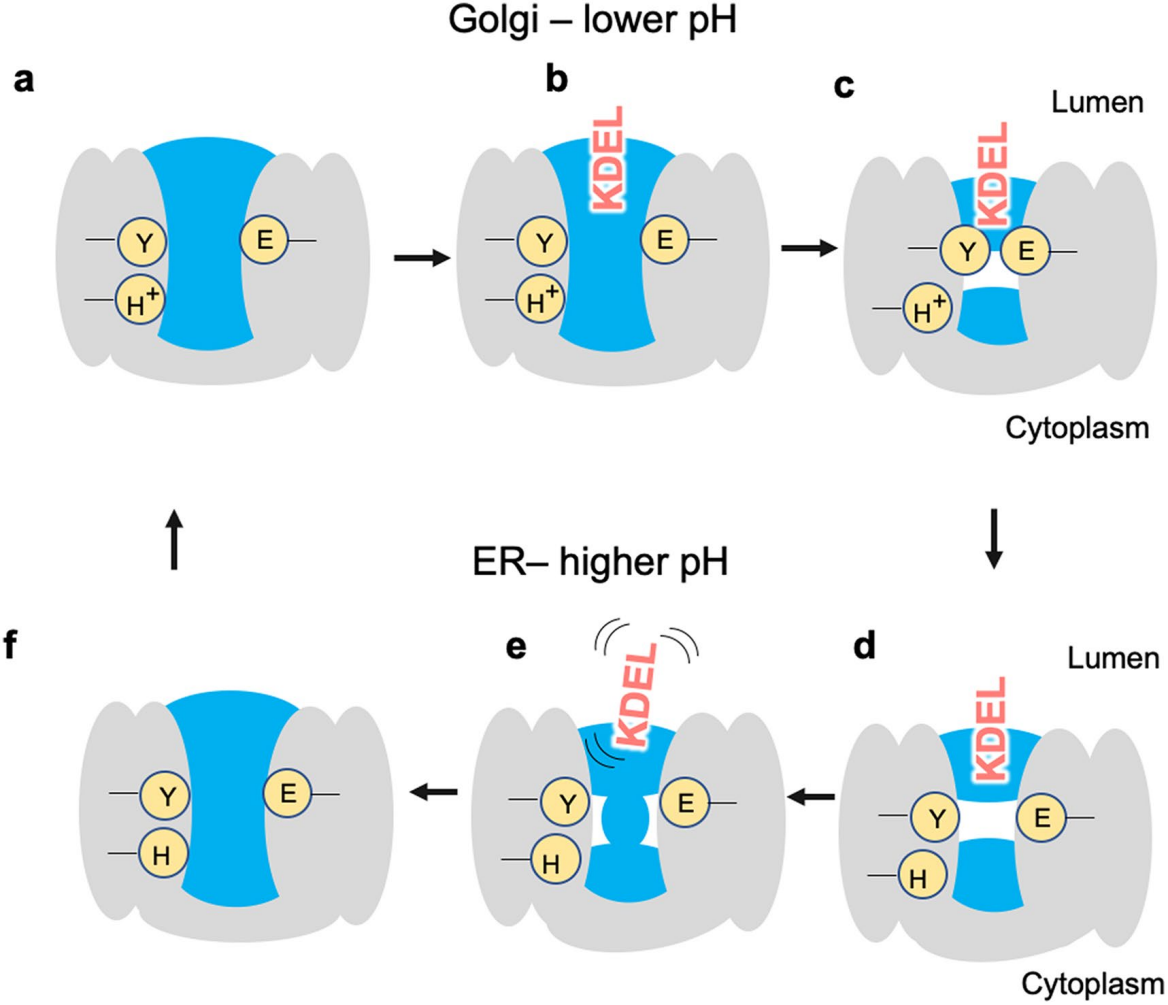
In the Golgi, the KDEL receptor is initially in its apo state (**a**), with the key histidine protonated and water present on both sides of E127/Y158. The KDEL-tagged substrate binds the receptor starting to trap waters as it enters (**b**). As binding completes some waters are cut from access to the lumen, the short hydrogen bond forms and the receptor undergoes other conformational changes to adopt the crystallographically observed KDEL-bound conformation (**c**). The KDEL receptor/KDEL-tagged substrate complex is then transferred to the ER where the higher pH causes deprotonation of the histidine. Without the protonated histidine, the short hydrogen bond is no longer maintained (**d**) and, the KDEL-tagged substrate is destabilized and dissociates (**e**). Since deprotonated histidine also favours a continuous water network, the receptor changes conformation and the water pocket is connected to bulk again (**f**).

What does all this mean for the function of the receptor? The receptor binds KDEL-tagged proteins in the Golgi where the pH is lower than in the ER (where the receptor releases its cargo). H12 has already been proposed to be the pH sensor^13^ and our observation here that a protonated H12 leads to a strengthening of the H-bond suggests that it likely plays a role in forming a tighter complex with the ERS. At neutral pH (as found in the ER), the short H-bond is weakened, and this facilitates the release of the KDEL-containing cargo protein. We also know from previous structural work, that in the KDEL-bound state, there is a different conformation of helix 7 which moves I147 out of the cytoplasmic pocket. Based on our observations of the hydrogen-bonded networks that connect the two water pockets either side of H12, we postulate that the protonation state of the receptor may also play a role in controlling the water-dynamics, which in turn might control the preferred conformational state of the receptor. Thus, the role of the short H-bond is complex. However, a mechanistic link between proton binding, water network rearrangements and structural stability are similar themes in secondary active transporters^24–26^ with which the KDELR shares the same topology and suggested evolutionary ancestry^27^. We summarize our results in Fig. 6.

## Conclusion

In this work we have performed extensive calculations that support the existence of a short H-bond in the KDEL receptor and characterize the influence this would have on receptor dynamics. Moreover, we have shown how the protonation state of a key histidine (H12) can dramatically affect the strength of that hydrogen bond. Via investigation of the reciprocal effect (of the short H-bond on the affinity of H12 for a proton) we also found a hydrogen-bonded water network that is broken in the presence of the short H-bond in the KDEL-bound state only and may have ramifications for the conformational dynamics of the receptor, something that we are currently further exploring.

## Methods

### QM/MM simulations

The KDEL-bound KDEL receptor (KDELR) and the Apo KDEL receptor were retrieved from the PDB (Apo: 6I6B; KDEL-bound: 6I6H) and embedded in a DMPC bilayer following a previously described protocol^28^. The QM region was defined as the three key residues (H12, Y158 and E127) and was described at the level of B97-3c^29^, whereas the qmcut was set to 3 nm to cover the whole protein. The force field amber 14SB^30^ was used to describe the protein and LIPID17^31^ was used to describe the lipid. The simulation was run with ambertools19^32^ in conjunction with ORCA 4.2.0^33^. For the free energy calculation, the collective variable chosen was the distance between the oxygen in the hydroxyl group of the Y158 and the closet oxygen in the carboxyl group of E127. 11 equally spaced windows from 2.4 to 3.4 Å with a restraint of 25 kcal/mol/Å were run with a 1 fs timestep for 20 ps. The free energy profile was constructed with WHAM 2.0.9.1.^34^.

### QM geometry optimisation

The coordinates of residues H12, E127 and Y158 were extracted from the crystal structure of both Apo (PDB: 6I6B) and the KDEL bound (PDB: 6I6H) KDELR. This very simplistic model was used as although one might assume that other nearby waters and protein residues might be important, the cooperative nature of conformational states means that it becomes totally arbitrary to decide at which point to cut-off the system. Thus, rather than make this arbitrary decision we examine this simple system. Note that in the original crystal structures the oxygen closest to the tyrosine OH is labelled OE2. To simplify the analysis here, we have relabelled that OE1 in this work. The N-termini of the three residues were capped with an acetyl group and the C-termini were capped with an N-methyl group. Hydrogen atoms were added to the crystal structure and different protonation states of the three residues were assigned using Maestro. The structure of the H12, E127 and Y158 complex, as well as each capped amino acid monomer and all the possible dimers (H12/E127; H12/Y158) were independently optimised with non-hydrogen atoms constrained using B3LYP together with the def2-TZVP^35^ basis set with DFT-D4^36^ for the empirical dispersion correction. Due to a known bug in the ORCA frequency calculations, the following flags (%freq Hess2elFlags[0] 0 Hess2elFlags[3] 0 end) were added as is suggested by the developer (https://orcaforum.kofo.mpg.de/viewtopic.php?f=26&t=5566). Vibrational frequency analysis was done at the end of optimisation using ORCA 4.2.0^33^. The CVBi^15^ and AIM analysis was performed with Multiwfn 3.7^37^.

### QM interaction energies

Single point energies were computed on the optimised structure with DLPNO-CCSD(T)/aug-cc-pVTZ^38^ at the level of normal PNO, with aug-cc-pVTZ/JK for RIJK acceleration and aug-cc-pVTZ/C^39^ for electron correlation calculations. The thermal correction for the Gibbs free energy was computed from the frequency calculations and corrected with quasi-harmonic correction^40^ and a zero-point energy (ZPE) scaling factor of 0.985^41^ using Shermo^42^ to 310 K. For complexes with more than one amino acid, the energy is corrected for by basis set superposition error (BSSE). BSSE is defined as half^43^ the BSSE energy. The interaction energy between E127(E) and Y158(Y) under the influence of the H12(H) is defined as

$$ G = G\left( {EYH} \right) - G\left( {EH} \right) - G\left( {YH} \right) + G\left( H \right) $$

where *G(EYH), G(EH), G(YH), G(H)* are the free energy of the geometry optimised E127, Y158, H12 complex, geometry optimised energy of the H12 in complex with E127 or Y158 and the energy of geometry optimised histidine, all with counterpoise corrected.

### QM proton transfer energy

The energy of transferring a proton from tyrosine to glutamate was computed at the level of PWPB95-D4/def2-QZVPP//B3LYP-D4/def2-TZVP^44^. The calculation was performed in the presence of three different protonation states of H12. During the geometry optimisation, the positions of all the non-hydrogen atoms were constrained and the bond between the proton and the closest oxygen was also constrained. The vibration mode corresponding to motion of the key proton was taken as the mode with the highest infrared intensity and confirmed with visual inspection using Gaussview 6.0. The ZPE corresponding to this mode was computed using Shermo^42^ .

### Protonation free energy calculations

A double bilayer box with KDEL-bound and Apo protein was constructed. Bilayers were placed 3 nm apart and solvated with TIP3P water and ions added up to 150 mM NaCl. The short hydrogen bond was maintained with a harmonic restraint joining the two oxygens in E127 and Y158 with restraint strength of 10,000 kJ/mol/nm. The equilibrium length is taken from the crystal structure. The topology for alchemical transformation was generated with pmx^45^, where the histidine in the KDEL-bound structure was transformed from HIP to HID, while the same histidine in the Apo state were transformed from HID to HIP. 21 equally spaced windows were used during the transformation, where the bonded, charge and vdw parameters were changed linearly at the same time. The simulations were run with Gromacs 2019.1^46^, with parameters taken from^47^. After the initial energy minimisation, a 200 ps simulation in the NVT ensemble were conducted, followed by a 1 ns run in the NPT ensemble. The production run was performed with replica exchange at an interval of 1 ps for 30 ns. The resulting energy files were analysed with alchemical exchange^48^ with the first 5 ns discarded as equilibration time.

### Grand canonical Monte Carlo (GCMC) simulations

The GCMC simulation were run with ProtoMS 3.4.0^49^. The protein system is generated as previously described by us^50^. The water pocket is defined as the bounding box encompassing the 12 waters in the crystal structure with a dimension of 43.6 × 29.8 × 75.3 Å^3^. 32 equally spaced Adams windows from − 17.0 to − 1.5 were used. Five repeats of 200 millions steps of production run were preceded with 15 million steps of insertion and deletion only equilibration and 5 million steps of full equilibration.

## Supplementary information

Supplementary Information 1.

## Data Availability

The data is available from the authors upon request.
