# Supplementary material for: The KDEL trafficking receptor exploits pH to tune the strength of an unusual short hydrogen bond
Source: Sci Rep. 2020 Oct 9;10:16903. doi: 10.1038/s41598-020-73906-3 (PMC7547670; doi:10.1038/s41598-020-73906-3)
Supplement: Supplementary file 1 — Supplementary Information 1. [file 41598_2020_73906_MOESM1_ESM.pdf]

Supplementary Information

The KDEL Trafficking Receptor Exploits pH to Tune the Strength of an Unusual Short Hydrogen Bond.

Zhiyi Wu, Simon Newstead* and Philip C. Biggin*.

Department of Biochemistry, South Parks Road, Oxford. OX1 3QU. UK.

*Email: Simon.newstead@bioch.ox.ac.uk and Philip.biggin@bioch.ox.ac.uk

SI Text 1. Establishing the position of the proton

It is well known that X-ray diffraction experiments often cannot resolve the precise location of hydrogen atoms and indeed for this reason they are omitted from the refined models. To characterize the nature of the hydrogen bond between E127 and Y158 and the influence of the nearby H12, we first needed to obtain precise positions of the hydrogen that would, under most conditions, be strongly associated with the hydroxyl oxygen of the tyrosine. Since, in this case our working hypothesis is that there is a particularly short hydrogen bond between the E127 and Y158, we do not know, *a priori*, which atom the hydrogen bond could be bonded to and there are three candidate oxygen atoms to consider; the hydroxyl oxygen of Y158 (labelled OH), and the two carboxy oxygens of E157 (labelled OE1 and OE2, see **Fig. 1** of the main manuscript). We thus set up geometry optimizations (**see Methods**) where a hydrogen atom is initially positioned next to one of these oxygens. This approach at least allows the coordinates to adopt a “correct from the quantum mechanical point of view” position of the hydrogens. In the Apo receptor, all hydrogens remain close to their original position after geometry optimization. In the KDEL-bound conformation, when the hydrogen is placed next to E127:OE1, which is the oxygen of the E157 closest to Y158, the position of the hydrogen converges during the optimization toward the oxygen of Y158 and indeed overlaps with the geometry optimized position of the hydrogen when it is initially placed next to the oxygen of Y158 (Y158:OH). Contrary to this, if the hydrogen is positioned closed to E127:OE2, the hydrogen remains bound to that oxygen. Thus, there are clear differences in the energetic landscape of the hydrogen in these different conformations.

To predict the most probable location of the hydrogen among these three oxygens, we computed the free energy of the geometry optimised structures. As is shown in **SI Table 1**, protonated Y158 in complex with the deprotonated E127 (E127···H—Y158) always has the lowest free energy compared to the two protonated forms of E127 (E127—HE1···Y158 and E127—HE2···Y158). To understand this further, we computed the surface (0.001 a.u. [14]) electrostatic potentials. As is shown in **SI Fig. 1**, only E127···H—Y158 shows good charge complementary, whereas both E127—HE1···Y158 and E127—HE2···Y158 have significant overlap between negative charge, which render them energetically less favourable.

Thus, all calculations are consistent with the expectation that the most favourable position for the proton in all states is close to the tyrosine oxygen.

SI Table 1. QM characterization of the E127:Y158 hydrogen bond of the KDEL receptor.

		Free energy (kcal/mol)	R(D-H)	R(H···A)	A(D-H···A)
	**KDEL-bound**				
**(HID12)**	E127···H—Y158	0.00	1.044	1.496	174.264
**(HID12)**	E127—HE2···Y158	46.48	0.995	2.150	145.370
**(HIE12)**	E127···H—Y158	1.64	1.029	1.510	174.352
**(HIE12)**	E127—HE2···Y158	48.23	0.997	2.148	145.528
**(HIP12)**	E127···H—Y158	0.00	1.058	1.482	173.371
**(HIP12)**	E127—HE2···Y158	43.93	0.991	2.166	144.051
	**Apo**				
**(HID12)**	E127···H—Y158	0.63	1.004	1.861	163.691
**(HID12)**	E127—HE1···Y158	11.29	1.039	1.803	174.172
**(HID12)**	E127—HE2···Y158	40.26	0.985	2.206	145.279
**(HIE12)**	E127···H—Y158	0.00	0.998	1.868	163.505
**(HIE12)**	E127—HE1···Y158	14.97	1.052	1.790	174.668
**(HIE12)**	E127—HE2···Y158	44.80	0.987	2.205	145.376
**(HIP12)**	E127···H—Y158	0.00	1.005	1.867	161.615
**(HIP12)**	E127—HE1···Y158	12.55	1.034	1.812	171.474
**(HIP12)**	E127—HE2···Y158	31.38	0.983	2.224	143.936

Free energy is computed at the level of D3LYP-D4/def2-TZVP. R(D-H) is the intramolecular distance between the donor heavy atom and the hydrogen. The free energy is normalized to the lowest value in each system. R(D-H) is the intramolecular distance between the donor heavy atom and the hydrogen. R(H···A) is the intermolecular distance between the hydrogen and the acceptor heavy atom. A(D-H···A) is the angle of the hydrogen bond along the donor heavy atom, the hydrogen and the acceptor heavy atom. HID12 indicates the system is optimised under a histidine which was protonated on the delta nitrogen, whereas HIE12 indicates that the histidine was protonated on the epsilon nitrogen. HIP12 means that both nitrogens were protonated. E127···H—Y158 stands for the hydrogen bond system between protonated Y158 and deprotonated E127, while E127—HE1···Y158 and E127—HE2···Y158 signifies the deprotonated Y158 hydrogen bonding to the E127 protonated on the E127:OE1 and E127:OE2 oxygen atoms respectively.

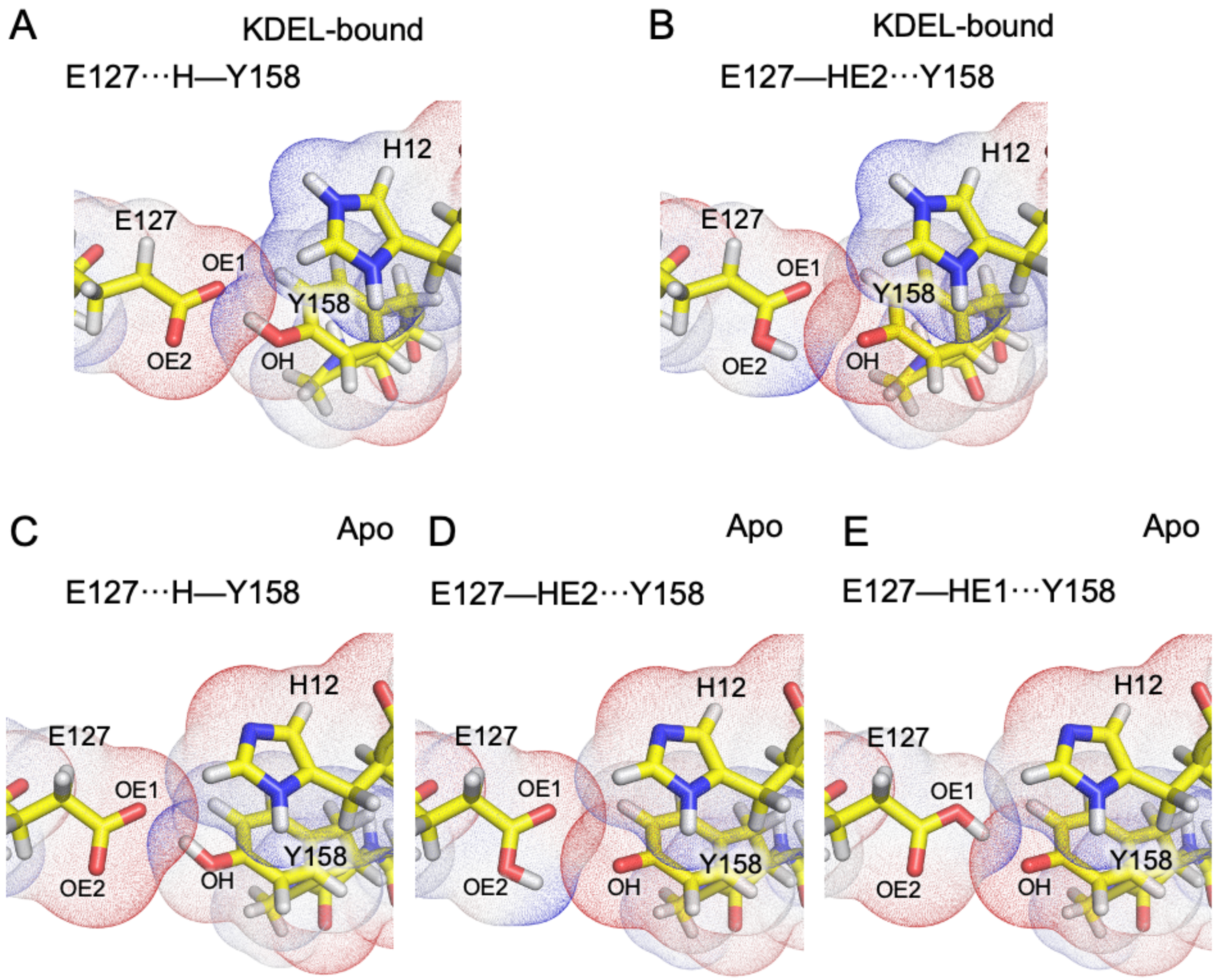

SI Figure 1. Electrostatic surface potentials. *The region of negative electrostatic potential is given in red, and the region of positive electrostatic potential is given in blue. In the KDEL-bound conformation (A, B), E127···H—Y158 has complementary charge overlap during the region of the hydrogen bond (A), whereas the region of overlap in the E127—HE2···Y158 system is comprised mainly of like (negative) charge (B). Favourable overlap is also observed in E127···H—Y158 in Apo conformation for both the E127···H—Y158 system (C) and the E127—HE1···Y158 system but not the E127—HE2···Y158 (D) system.*

SI Text 2. Details on the Quantum Theory of Atoms in Molecules (QTAIM)

According to Bader's the quantum theory of atoms in molecules (QTAIM) theory, topology analysis of the electron density, (ρ_r) and its Laplacian can be used to give a quantitative description of the hydrogen bond system. For a bond to present, an atomic bond path has to link the the two atoms that form the bond and a bond critical point (BCP) has to be present on this path[1,2]. In all hydrogen bond systems, an atomic bond path was found between the proton and the hydrogen bond acceptor and a BCP is present on this path. Table 2 of the main manuscript lists some important topological parameters for this BCP, including the total energy density H(r)[3], which is the sum of Lagrangian kinetic energy G(r) and potential energy density V(r)[4], the V(r)/G(r), H(r)/ρ(r), where ρ(r) is electron density. The strength of the hydrogen bond can be inferred from H(r), where Rozas et al[5] proposed that a weak hydrogen bond shows $H(r) >= 0$ and a strong hydrogen bond shows $H(r) < 0$.

The hydrogen bonds formed in the KDEL-bound states between the Y158H and E127 can be classified as a strong hydrogen bond while the hydrogen bonds in the apo state are classified as a weak hydrogen bond. The strongest hydrogen bond is observed when the histidine is protonated (HIP) in the KDEL-bound state. Espinosa et al.[4] purposed both H(r)/ρ(r) and V(r)/G(r) can serve as an indication of the bond strength. H(r)/ρ(r) is also commonly called the bond degree (BD) and a smaller value indicates a stronger bond. Similar to BD, the smaller the V(r)/G(r), the stronger the bond. The rank of hydrogen bond strength is consistent with all other analysis where the hydrogen bond is stronger in KDEL-bound state compared with the apo state and the hydrogen bond in the KDEL-bound state under protonated histidine (HIP) is the strongest.

SI Table 2. RMSF (Å) of H12 during alchemical transformations.

	HID	HIP
KDEL-bound (SHB)	0.54 ± 0.05	0.41 ± 0.01
KDEL-bound	0.60 ± 0.02	0.50 ± 0.01
Apo	0.54 ± 0.01	0.58 ± 0.01

References

1 Bader, R. F. W. *Atoms in molecules: A quantum theory*. (Clarendon Press, 1990).
2 Popelier, Paul L. A. *Atoms in molecules: An introduction*. (Prentice Hall, 2000).
3 Cremer, D. & Kraka, E. Chemical bonds without bonding electron density — does the difference electron-density analysis suffice for a description of the chemical bond? *Ange.Chem. Int. Ed.* **23**, 627-628, (1984).
4 Espinosa, E., Alkorta, I., Elguero, J. & Molins, E. From weak to strong interactions: A comprehensive analysis of the topological and energetic properties of the electron density distribution involving X–H···F–Y systems. *J. Chem. Phys.* **117**, 5529-5542, (2002).
5 Rozas, I., Alkorta, I. & Elguero, J. Behavior of ylides containing N, O, and C atoms as hydrogen bond acceptors. *J. Am. Chem. Soc.* **122**, 11154-11161, (2000).
